# Targeting and monitoring ovarian cancer invasion with an RNAi and peptide delivery system

**DOI:** 10.1073/pnas.2307802121

**Published:** 2024-03-04

**Authors:** Liangliang Hao, Natalie Boehnke, Susanna K. Elledge, Nour-Saïda Harzallah, Renee T. Zhao, Eva Cai, Yu-Xiong Feng, Sofia Neaher, Heather E. Fleming, Piyush B. Gupta, Paula T. Hammond, Sangeeta N. Bhatia

**Affiliations:** ^a^Koch Institute for Integrative Cancer Research, Massachusetts Institute of Technology, Cambridge, MA 02139; ^b^Department of Chemical Engineering and Materials Science, University of Minnesota Twin Cities, Minneapolis, MN 55455; ^c^Harvard University–Massachusetts Institute of Technology Division of Health Sciences and Technology, Institute for Medical Engineering and Science, Massachusetts Institute of Technology, Cambridge, MA 02139; ^d^Department of Biology, Whitehead Institute for Biomedical Research, Cambridge, MA 02142; ^e^Department of Electrical Engineering and Computer Science, Massachusetts Institute of Technology, Cambridge, MA 02139; ^f^Naveris, Waltham, MA 02451; ^g^Institute for Soldier Nanotechnologies, Massachusetts Institute of Technology, Cambridge, MA 02139; ^h^Department of Chemical Engineering, Massachusetts Institute of Technology, Cambridge, MA 02139; ^i^Marble Center for Cancer Nanomedicine, Massachusetts Institute of Technology, Cambridge, MA 02139; ^j^Broad Institute of Massachusetts Institute of Technology and Harvard University, Cambridge, MA 02142; ^k^Department of Medicine, Brigham and Women’s Hospital, Boston, MA 02115; ^l^Wyss Institute for Biologically Inspired Engineering, Harvard University, Boston, MA 02115; ^m^HHMI, Massachusetts Institute of Technology, Cambridge, MA 02139

**Keywords:** theranostics, proteases, RNAi delivery, Layer-by-layer assembly, synthetic biomarker

## Abstract

RNA interference (RNAi) therapeutics are an emerging class of medicines that selectively target mRNA transcripts to silence protein production and combat disease. Despite the recent progress, a generalizable approach for monitoring the efficacy of RNAi therapeutics without invasive biopsy remains a challenge. Here, we describe the development of a self-reporting, theranostic nanoparticle that delivers siRNA to silence a protein that drives cancer progression while also monitoring the functional activity of its downstream targets. Our therapeutic target is the transcription factor SMARCE1, which was previously identified as a key driver of invasion in early-stage breast cancer. Using a doxycycline-inducible shRNA knockdown in OVCAR8 ovarian cancer cells both in vitro and in vivo, we demonstrate that SMARCE1 is a master regulator of genes encoding proinvasive proteases in a model of human ovarian cancer. We additionally map the peptide cleavage profiles of SMARCE1-regulated proteases so as to design a readout for downstream enzymatic activity. To demonstrate the therapeutic and diagnostic potential of our approach, we engineered self-assembled layer-by-layer nanoparticles that can encapsulate nucleic acid cargo and be decorated with peptide substrates that release a urinary reporter upon exposure to SMARCE1-related proteases. In an orthotopic ovarian cancer xenograft model, theranostic nanoparticles were able to knockdown SMARCE1 which was in turn reported through a reduction in protease-activated urinary reporters. These LBL nanoparticles both silence gene products by delivering siRNA and noninvasively report on downstream target activity by delivering synthetic biomarkers to sites of disease, enabling dose-finding studies as well as longitudinal assessments of efficacy.

## Significance

This study reports the development of a theranostic nanoparticle that delivers siRNA to silence the transcription factor SMARCE1 and monitors efficacy by reporting on downstream protease activity through urine detection. SMARCE1 promotes dissemination of ovarian cancer through regulating the expression of secreted proteases that degrade the ECM. Layer-by-layer nanoparticles enable integration of distinct chemical cargo such as nucleic acids directed to the cytoplasm and peptides directed to the tumor microenvironment on a single platform. The highly modular system provides the ability to monitor enzymatic profiles noninvasively, a strategy that could be employed to detect changes in protease activity and correlate them with disease progression and treatment response. Continued development of this platform will offer personalized treatment regimens in precision oncology.

RNA-based therapeutics have proven to be powerful modalities for treating disease through manipulation of protein production. Currently, four types of RNA therapies have been approved by the FDA, including antisense oligos (ASO) to sterically block mRNA translation, short-interfering RNA (siRNA) and microRNA (miRNA) to degrade mRNA (known as RNA interference, RNAi), RNA aptamers to bind to molecules, and mRNA to induce expression of a protein, exemplified by the widespread use of SARS-CoV-2 mRNA vaccines developed during the COVID-19 pandemic (1–4). In the context of cancer, both mRNA and RNAi therapies are being developed and tested in multiple clinical trials with many other RNA-based therapies in development (1–4). RNAi in particular is an intervention that can alter expression of essential oncogenic pathway transcription factors that regulate many cancers but that have been difficult to target with small molecule drugs (5, 6). Despite the great promise of RNAi therapies, there are many hurdles that have limited their success in the clinic. In particular, evaluating treatment efficacy is difficult without performing invasive biopsies or having access to a circulating or excreted biomarker to quantify. With these issues in mind, and due to the complications inherent in the in vivo delivery and pharmacodynamics of RNA agents, effective use of RNAi interventions will benefit from a self-reporting readout to inform dosing regimens and therapy selection. These decisions are time-sensitive and impactful, given that patient outcomes are dramatically enhanced when early intervention is successful (7, 8). Therefore, there is a need to develop RNAi administration mechanisms that both deliver RNA and report on its pharmacodynamic efficacy in a timely manner.

For the majority of approved RNA therapies, successful delivery and knockdown has been validated by evaluation of serum/urinary markers or by indirectly looking at disease severity. In the RNAi therapeutic space, four siRNA therapies have been approved, to date (4). In the case of Alnylam’s patisiran, an RNAi encapsulated nanoparticle approved in 2018 to silence liver transthyretin expression in polyneuropathy patients, pharmacodynamic analysis was evaluated by reduced serum levels of the silenced target gene (9). Similar readouts were leveraged by other approved RNAi drugs, such as lumasiran, givosiran, and inclisiran (2, 4, 9–12). In many cancer contexts, ideal targets are intracellular and cannot be detected in blood nor by a directly downstream secreted biomarker. Thus, for most targeted oncogenes, the only readout of efficacy is decrease in disease burden, which can require long time scales to manifest and not give direct insight into downstream effects. In an effort to overcome this limitation, some current RNA cancer therapy candidates utilize conventional cancer serum biomarkers (2, 4, 9–14) or, more recently, circulating tumor DNA (ctDNA) (15) as surrogate silencing efficacy readouts. While promising strategies, both suffer from signal heterogeneity, low sensitivity, and lack of faithful mapping to tumor burden (13, 16). As an alternative, multiple groups have designed synthetic biomarkers to detect effective delivery. Gambhir and others have developed small circularized DNA fragments that produce biomarkers or therapeutic enzymes in the presence of tumor-specific promoters but have not applied this strategy to siRNA therapy (17, 18). The Anderson group and others have developed methodology to optimize the delivery of siRNA-bearing nanoparticles that target factor VII, a secreted protein made in the liver and detected in the blood (19–21). While this method has proved useful for optimization of particular formulations, as illustrated during the early development of patisiran (22), it cannot accurately recapitulate the efficacy of other nonsecreted siRNA targets in vivo, nor delivery to organs other than the liver—where factor VII is synthesized. Our group and others have reported a growing panel of sense-and-report diagnostic platforms based on the design of activity-based nanosensors that utilize peptide cleavage by proteases to produce urinary synthetic biomarkers (23). Proteases are widely dysregulated in cancer and as such can provide more sensitive detection of tumor burden than canonical blood-based cancer biomarkers (24). Therefore, leveraging readouts that report on protease activity may serve to assay efficacy changes that arise with knockdown of cancer targets. Moreover, incorporating both therapeutic and diagnostic elements into a theranostic module could allow for simultaneous treatment and monitoring of intervention efficacy and thus inform treatment teams as they aim to optimize clinical outcomes.

**Fig. 1.**
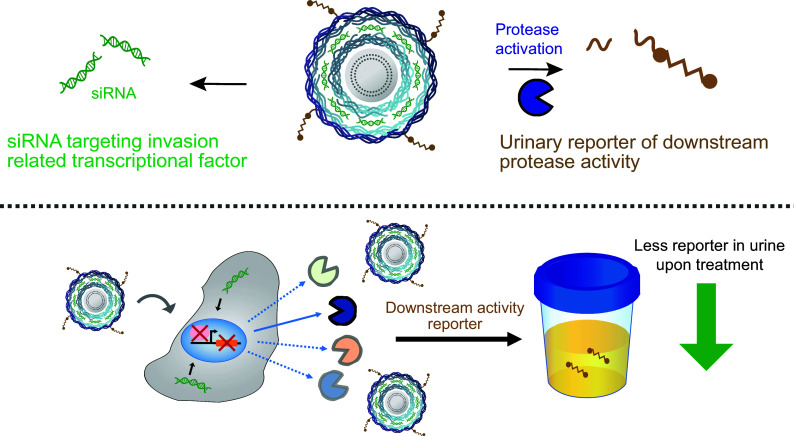
Overview of the self-reporting RNAi delivery system for simultaneous RNAi treatment and monitoring.

In order to deliver siRNA and report on downstream activity in a single entity, we sought out a modular and functionalizable delivery system. Recent advances in layer-by-layer nanoparticle (LbL NP) technology using electrostatic assembly of oppositely charged polyelectrolytes allow for loading a high density of siRNA molecules, extended particle circulation times, and enhanced therapeutic delivery (25, 26). In addition, these modular particles allow for complex surface functionalization through adsorption and click chemistry (27, 28). We recently collaborated to produce LbL NPs that can deliver siRNA and simultaneously report on an unrelated protease activity via their surface functionalized biosensors (28). Building on these collective approaches, in the present study, we have developed a self-reporting siRNA delivery nanoparticle that can be utilized to target a transcription factor in vivo and report on its efficacy based on changes in downstream protease activity (Fig. 1). We applied this theranostic LbL NP to target ovarian cancer, which remains the most lethal gynecologic cancer with poor prognosis and treatment outcomes (29). We selected the transcription factor SMARCE1 as our candidate therapeutic target, which was previously identified as a key driver of invasive progression of early-stage breast cancer (30). SMARCE1 is a master regulator of genes encoding proinvasive proteases that are required to degrade the basement membrane (30–32). We first demonstrate that similar to breast cancer, shRNA-mediated knockdown of SMARCE1 in ovarian cancer leads to decreased tumor growth by downregulation of invasive proteases. We report the concentric self-assembly of LbL NPs that encapsulate a high weight percentage of SMARCE1 siRNA and include an external layer of tumor targeting and synthetic biosensing peptides. After accumulation at the tumor site, siRNA is delivered intracellularly to knockdown SMARCE1. Additionally, the synthetic biomarkers are released after cleavage by proteases downstream of SMARCE1 in the tumor microenvironment. The liberated peptide fragment circulates and is concentrated by the kidney for urinary detection. These multifunctional theranostic particles resulted in SMARCE1 knockdown in vivo that correlated with a reduction in the amount of urine reporters produced when downstream protease activity was blunted following siRNA treatment.

## Results

### SMARCE1 Expression Correlates with Survival Outcome in Patients with Early-stage Ovarian Cancer.

**Fig. 2.**
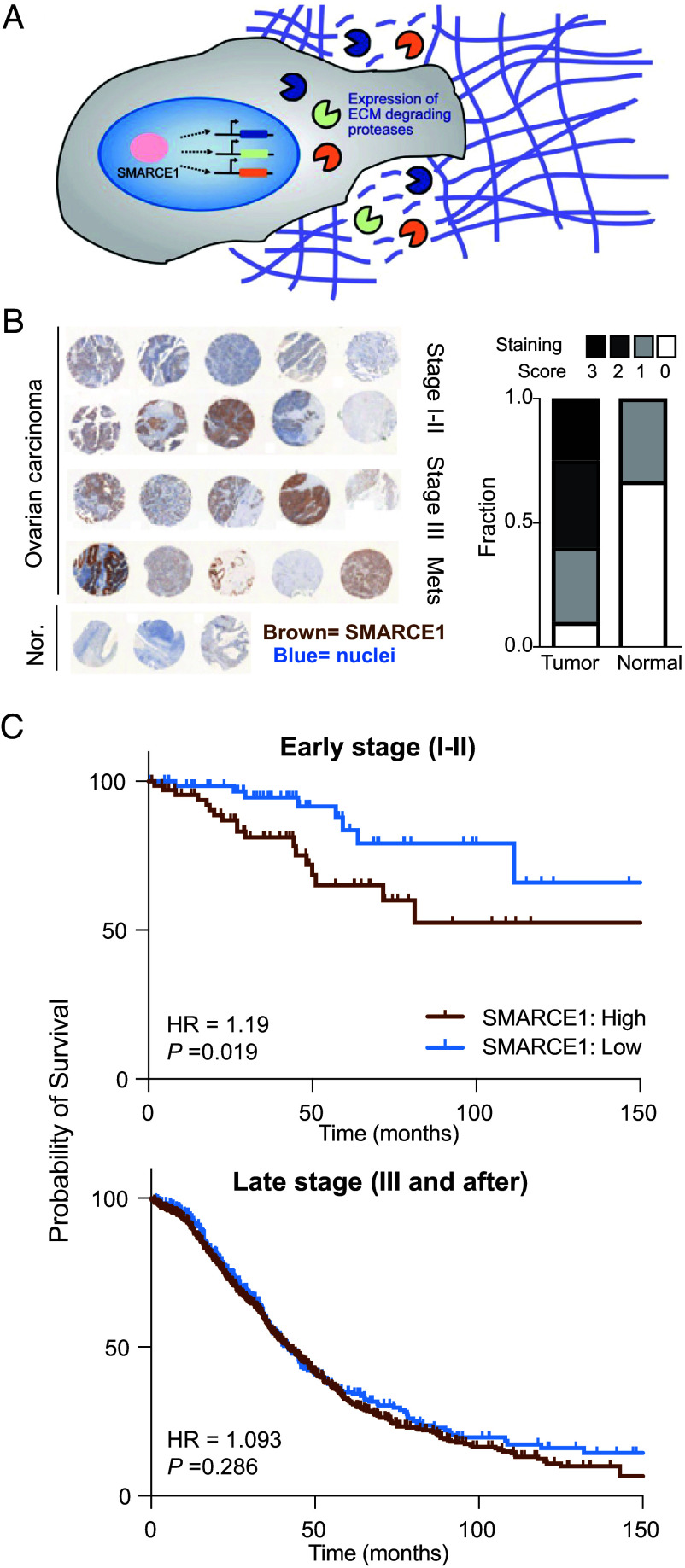
SMARCE1 expression levels correlate with survival in ovarian cancer. (*A*) Schematic exhibiting SMARCE1 regulation of invasion by inducing expression of ECM-degrading proteases30. (*B*) Human tissue biopsies from in situ and invasive ovarian cancers were immunohistochemically stained for SMARCE1 protein expression (*Left*). The staining intensities (*Right*) were scored and quantified by a blinded pathologist, where 3 was assigned to the highest level of staining (dark brown) and 0 for no visible staining. (*C*) Ovarian cancers (Kaplan–Meier Plotter database, ovarian cancer, see *Materials and Methods*) were analyzed for overall survival. Survival curves in ovarian cancer patients stratified into tertiles (high, low) were plotted based on tumor SMARCE1 expression. SMARCE1 expression was examined in a cohort of patients with early-stage ovarian tumors (stage I or II; n = 179) (*Top*) or late-stage ovarian tumors (stages III and IV; n = 1,268) (*Bottom*). Hazard ratios (HRs) and *P* values were determined with the log-rank statistical test.

SMARCE1 has been identified as a clinically relevant factor that promotes the invasive progression of early-stage tumors (30, 31, 33). In breast cancer, SMARCE1 is required for invasion of ductal carcinoma in situ (DCIS) through basement membrane by up-regulating multiple ECM-degrading proteases including collagen-degrading matrix metalloproteinases (MMPs) and serine proteases such as urokinase plasminogen activator (PLAU) that degrade laminin, another main component of basement membrane (30) (Fig. 2*A*). However, the functional contribution of SMARCE1 to the invasion of ovarian cancer and its regulatory impact on proteolytic activity remain understudied. We performed immunostaining for SMARCE1 protein in human ovarian cancer tissue microarrays (Fig. 2 *B*, *Left*) and observed that SMARCE1 was not detectable or expressed at very low levels in normal tissue and early-stage ovarian cancer. However, in tumor samples from late-stage ovarian cancer patients (III, Mets), SMARCE1 expression increased and was highest in tumors invading into regional lymph nodes and distant organs (Fig. 2 *B*, *Right* and *SI Appendix*, Fig. S1). Next, we mined existing clinical datasets to investigate any clinical correlation between SMARCE1 expression and probability of survival. The overall survival of ovarian cancer patients with high expression levels of SMARCE1 was more likely to show relapse with metastases over a follow-up period of more than 15 y [n = 1,435, hazard ratio (HR) = 1.19, *P* = 0.019] (*SI Appendix*, Fig. S2). In particular, patients with early-stage (I and II) ovarian tumors expressing high levels of SMARCE1 showed significantly lower probability of survival (n = 179, HR = 1.19, *P* =0.019) (Fig. 2 *C*, *Top*). In contrast, SMARCE1 expression was not predictive of survival for patients diagnosed with later-stage ovarian cancer tumors (stages III and IV) (n = 1,268, HR = 1.093, *P* = 0.286) (Fig. 2 *C*, *Bottom*). This finding is consistent with previous reports on correlations between SMARCE1 expression and ovarian cancer patient survival using a separate patient database (30).

### SMARCE1 Promotes Ovarian Cancer Invasion and Distant Metastasis In Vitro and In Vivo.

**Fig. 3.**
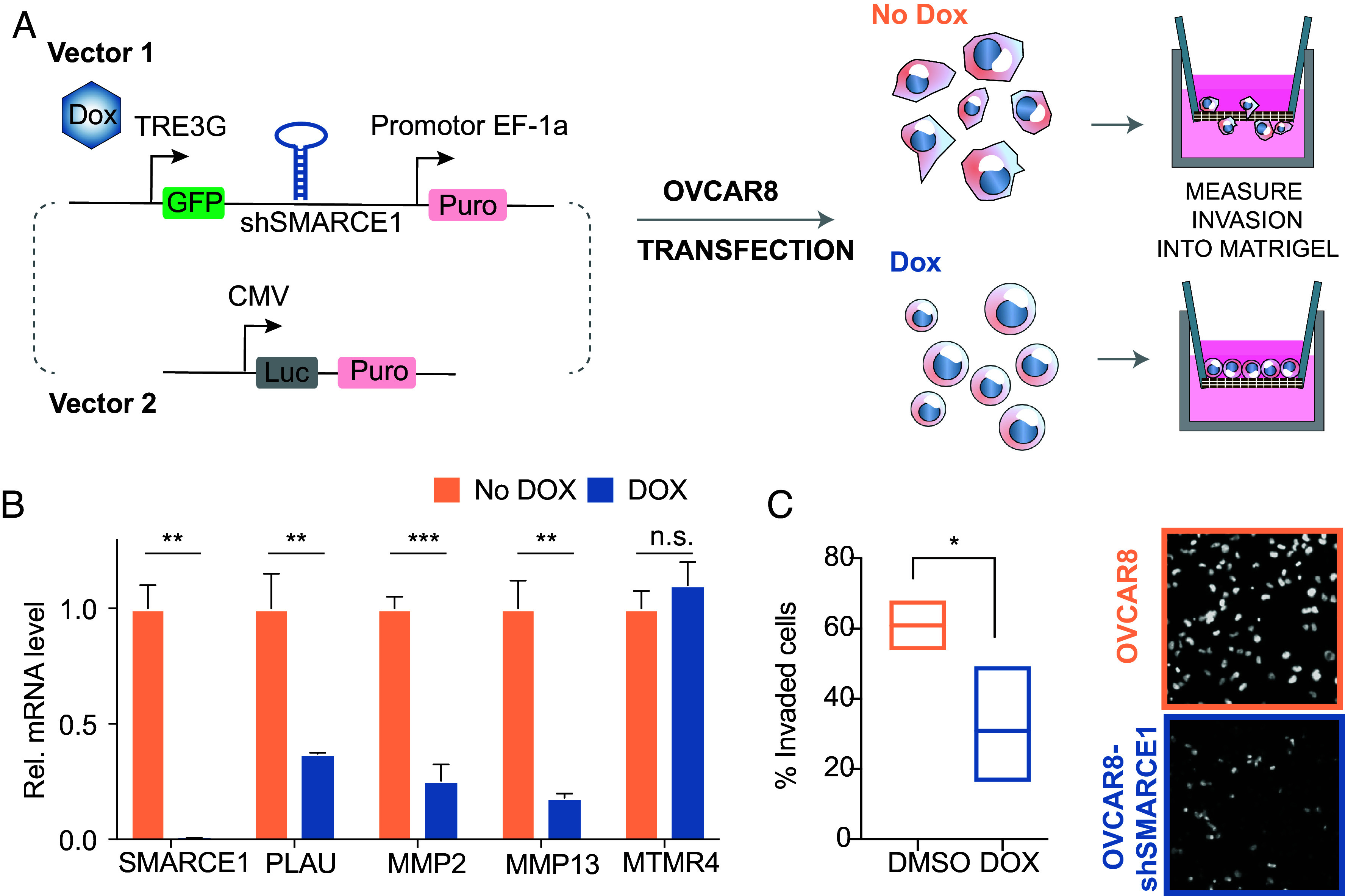
Knockdown of SMARCE1 in ovarian cancer cells reduces the expression of ECM invasion module genes and interferes with cell invasion in vitro. (*A*) Engineered system for doxycycline (DOX) inducible SMARCE1 knockdown in the OVCAR8 cell line. The first vector controls the expression of SMARCE1 shRNA under the DOX sensitive promoter TRE3G. A second vector induces stable expression of luciferase. Both vectors were transfected into the OVCAR8 cell line. Transfected cells were subsequently assayed in an ECM migration assay and migratory cells were quantified with and without DOX treatment. (*B*) Quantitative PCR analysis of expression of ECM invasion module genes and an unrelated module gene (MTMR4) in OVCAR8 cells treated with or without doxycycline to induce SMARCE1 shRNA expression. Gene expression is normalized to 18S RNA and plotted as fold change relative to the control cell line (n = 3 wells per condition, each well has three technological replicates, mean ± SEM, two-tailed Student’s *t* test, **P < 0.01). (*C*) Cell migration analysis using the Matrigel invasion chamber for native OVCAR8 cells compared to transduced and dox treated OVCAR8-shSMARCE1 cells. Invaded cells were stained and quantified (n = 6 wells per cell type, each well has four regions imaged, mean ± SEM, two-tailed Student’s *t* test, *P < 0.05, Scale bar, 50 µm).

We next investigated the functional impact of SMARCE1 in ovarian cancer progression. We engineered the invasive OVCAR8 cancer cell line with silenced SMARCE1 (OVCAR8-shSMARCE1) by stably expressing shRNA to inhibit SMARCE1 expression (Fig. 3*A*). Doxycycline-triggered SMARCE1 knockdown significantly reduced the expression of genes of proteases in the ECM invasion module, namely MMP2, MMP13, and PLAU, whereas it had no effect on the expression of a random control gene (Fig. 3*B* and *SI Appendix*, Fig S3). Given that up-regulated protease activities can result in invasive progression, we then seeded engineered and native OVCAR8 cells in the Matrigel invasion chamber to visualize cell migration. 60% of SMARCE1 expressing OVCAR8 cells passed through basement membrane layer, whereas only 30% of cells with SMARCE1 knockdown (OVCAR8-shSMARCE1) showed capability of invasion (Fig. 3*C*).

**Fig. 4.**
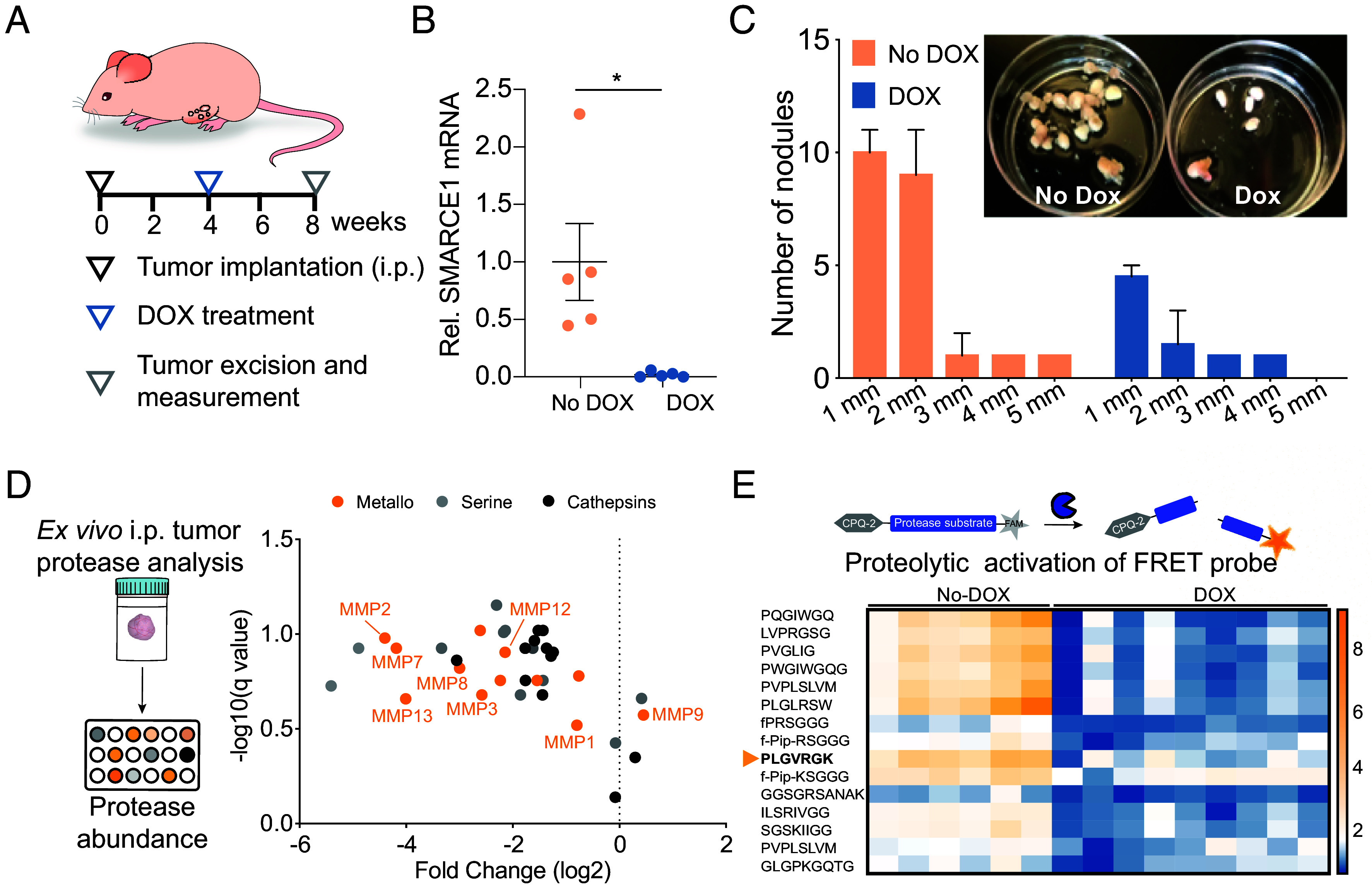
SMARCE1 is required for cancer cell invasion through regulation of protease expression and activity in vivo. (*A*) Schematic of the inducible orthotopic ovarian cancer model. (*B*) Quantitative PCR analysis of SMARCE1 gene expression in tumor nodules extracted from the intraperitoneal space of animals injected with OVCAR8-shSMARCE1 and fed with or without DOX chow diet for 4 wk. Gene expression is normalized to 18S RNA control and plotted as fold change relative to the control animals (n = 6; mean ± SEM, two-tailed Student’s *t* test, *P < 0.05). (*C*) Size distribution of tumor nodules retrieved from the intraperitoneal space of animals 4 wk after feeding of chow diet (n = 5 per group; Scale bar, 5 mm). Image shows a representative animal from each group. (*D*) Proteome Profiler™ Human Protease/Protease Inhibitor Array analysis quantifying protease expression from tumor nodules extracted from animals injected with OVCAR8-shSMARCE1 and fed the DOX chow diet. Protease abundance is plotted as fold change relative to the control animals not fed with DOX (n = 5 animals per group). Each dot represents a different protease and classes are color coded (metalloprotease, orange; serine protease, gray; cathepsin protease, black). Q values were calculated by Student’s *t* tests. (*E*) Schematic showing FRET-based protease substrate cleavage in response to treatment of tumor tissue lysates collected from animals injected with OVCAR8-shSMARCE1 and fed with or without DOX chow diet. The cleavage velocity of a substrate (concentration: 6 μM) by the tumor tissue lysates was plotted in the heatmap. Each column represents one animal (n = 9 for animals on the DOX diet, n = 6 for control group of animals on a standard diet). PLGVRGK marks a metalloprotease-sensitive substrate reporter sequence typically cleaved at elevated levels in a range of tumor models (24).

In light of the findings that SMARCE1 promotes ovarian cancer invasion in vitro, we next assessed the in vivo function of SMARCE1 by using an orthotopic mouse model of OVCAR8-derived tumor formation. In this model, disseminated tumors are formed by introducing OVCAR8-shSMARCE1 cells that stably express luciferase via intraperitoneal (i.p.) implantation in female NCr Nude mice (Fig. 4*A*). The injected cells form disseminated tumor nodules on the timescale of weeks and the mice eventually accumulate ascites (timescale of months), which mimics disease progression occurring in human ovarian cancer patients. When fed with chow diet containing doxycycline (DOX), production of shSMARCE1 silences the expression of SMARCE1 (Fig. 4*B*). Four weeks after being put on DOX-containing diet, mice showed significant regression of total tumor burden, reflected in decreased nodule number and median nodule diameter (Fig. 4*C*). To further explore whether SMARCE1 was required for an invasiveness program, we performed tail-vein injections of OVCAR8-shSMARCE1 cells and longitudinally monitored lung tumor growth via IVIS imaging. Three weeks after injection, lung tumor burden was ∼10-fold lower in mice injected with SMARCE1-inhibited cancer cells on the DOX diet compared with mice injected with cells on the standard diet (*SI Appendix*, Fig. S4).

### Inhibition of SMARCE1 Interferes with Downstream Protease Activity.

After confirming that knockdown of SMARCE-1 results in decreased tumor burden, we sought to determine the protease activity landscape following knockdown, to evaluate whether protease sensing could be leveraged as a readout. We collected intraperitoneal tumor nodules from the orthotopic mouse model derived by the OVCAR8-shSMARCE1 cells. Total RNA was extracted from tumor nodules in animals on chow diet with (SMARCE1 knockdown) or without DOX (SMARCE1-expressing). We then assayed 92 tumor metastasis-associated genes via quantitative PCR analyses (*SI Appendix*, Fig. S5 and Dataset S1). A large portion of this panel of genes encoded for multiple secreted proteases that degrade collagen and laminin, the two main components of the basement membrane. Among these proteases were collagen-degrading matrix metalloproteinases (MMPs) including MMP1, 2, 7, 9, and 13. In addition, this panel includes ECM components that stimulate cancer cell invasion, such as collagens (e.g., COL1A1, COL4A1), the extracellular matrix glycoproteins laminins (e.g., LAMA1, LAMC1), fibronectin (FN1), and SPARC, among others. The module also included cell adhesion molecules such as integrins (e.g., ITGA1) and catenins (CTNNA1) that have been previously implicated in invasion. To confirm that tumor-associated proteases are decreased at the protein level upon SMARCE1 knockdown, we simultaneously measured expression of 35 proteases in a single sample using multiplexed western blots (Fig. 4*D* and Dataset S2A). Consistent with the results of the quantitative PCR, we observed a global inhibition of protease expression in orthotopic tumors with SMARCE1 knockdown, in particular across a wide range of MMPs. To look more directly at protease activity, rather than abundance, we incubated tumor tissue lysates with a small FRET-based library of broad MMP protease substrates that dequench fluorescence upon proteolytic cleavage (Fig. 4*E*, *SI Appendix*, Table S2, and Dataset S2B). In nine animals with SMARCE1 knockdown, we show that the activities of proteases are reduced globally compared with control animals with SMARCE1 expression. We chose to use the PLGVRGK substrate as a candidate self-reporting readout sensor to detect broad MMP protease activity because this peptide has shown efficacy in tracking progression of ovarian cancer models (24) and exhibited strong reduction in cleavage signal following SMARCE1 knockdown (*SI Appendix*, Fig. S6).

### Theranostic Nanoparticle-mediated Knockdown of SMARCE1 In Vivo Can Be Monitored via Protease-activated Urinary Reporter.

**Fig. 5.**
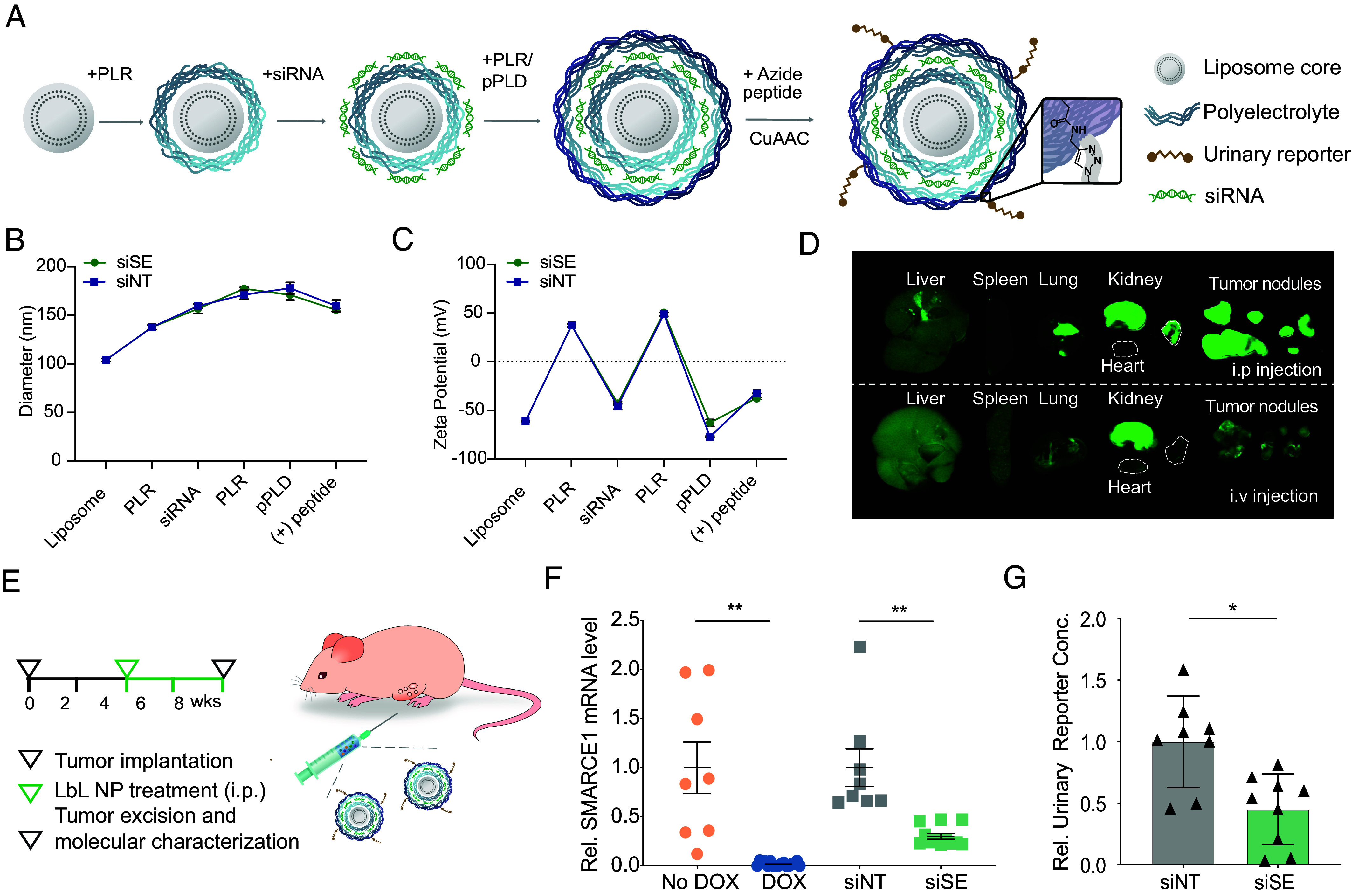
Theranostics interfere with ovarian cancer invasion through knockdown of SMARCE1 that was monitored via a protease-activated urinary reporter. (*A*) Schematic display of concentrically self-assembled LbL NPs that encapsulate SMARCE1 siRNA while also carrying a synthetic biosensing peptide on the surface that is cleaved into a urinary reporter upon exposure to specific proteases that are responsive for SMARCE1 knockdown. The size (*B*) and zeta potential (*C*) of the LbL NPs were monitored during synthesis using dynamic light scattering. Error bars represent SD of triplicate measurements. (*D*) Tissue distribution of siRNA-SMARCE1 LbL NPs with Cy7-labeled liposomal cores injected through i.p. and i.v. administration. (*E*) Theranostic LbL NPs were i.p and injected to the orthotopic ovarian cancer model to provide an siRNA dosage of 0.5 mg/kg to silence SMARCE1 in vivo. After four weekly doses, SMARCE1 expression was quantified by qPCR of excised tumors. (*F*) SMARCE1 expression was quantified by qPCR of excised tumors. Animals fed with DOX chow diet were assayed as positive control. siNT refers to LbL NPs carrying nontargeting control siRNAs; siSE refers to LbL NPs carrying siRNAs targeting SMARCE1. Both particle formulations carry the MMP-responsive urinary reporter (n = 8 for No DOX and siNT groups, n = 9 for DOX and siSE groups; mean ± SEM, two-tailed Student’s *t* test, **P < 0.01). (*G*) Analysis of reporters in mouse urine samples collected 1 h after final injection by ELISA.

Although SMARCE1 plays an essential role for the escape of tumor cells from the normal tissue architecture into the surrounding matrix, the potential of SMARCE1 inhibition to prevent tumor progression has yet to be explored due to the lack of drugs targeting transcription factors. In addition, there is a need for tools to provide information about the proteolytic tumor microenvironment, which is correlated with patient prognosis. Herein, we combined the multicomponent features and tumor-targeting capabilities of layer-by-layer assembly with functional biosensing peptides to create a class of theranostics. LbL NPs are modular drug delivery vehicles that incorporate multiple functional materials through sequential deposition of polyelectrolytes onto charged nanoparticle cores (Fig. 5*A*). The particles are carefully crafted by utilizing a liposome core, followed by layering of poly-l-arginine (PLR) designed for endosomal escape (34), siRNA, PLR, and propargyl poly-l-aspartic acid (PLD) for surface functionalization. In this study, LbL nanoparticles are engineered to encapsulate gene-silencing siRNAs against SMARCE1 and a synthetic biosensing peptide on the surface that is cleaved into a urinary reporter upon SMARCE1 knockdown (35, 36). The size, uniformity, and charge were tracked during LbL synthesis of the nanoparticles carrying siRNAs against SMARCE1 (siSE-LbL) or the nontargeting control siRNAs (siNT-LbL). Collectively, the data demonstrated the controlled growth of both types of particles: The Z-average diameter increased from 104 ± 1.5 nm (plain liposome core) to 155 ± 1.4 nm (siSE-LbL) and 159 ± 5.9 nm (siNT-LbL), respectively (Fig. 5*B*), consistent with our observations with previous, similar LbL NP systems (28). The sequential charge reversal, especially a reduction in zeta potential upon siRNA loading (from 37.3 ± 1.6 mV to −42.8 ± 1.7 mV for siSE-LbL, and 37.3 ± 1.6 mV to −45.8 ± 2.9 mV for siNT-LbL), indicated successful LbL assembly (Fig. 5*C*). The polydispersity (PDI) values stayed within 0.25 throughout the synthesis (*SI Appendix*, Fig. S7*A*). To enable the diagnostic capability, LbL NPs were modified on the surface by copper-catalyzed click chemistry-mediated covalent conjugation of the PLGVRGK MMP-activated reporter peptide. The outer polyelectrolyte layer containing propargyl-poly-L-aspartic acid (pPLD) was used to covalently attach the azide-containing biosensor peptide using copper-catalyzed azide-alkyne click chemistry (CuAAC) (Fig. 5*A*). We expect this surface reporter peptide to be cleaved by extracellular proteases (rapidly, <1 h) prior to the internalization of the LbL particle (>4 h). We quantified the purification kinetics in order to determine the encapsulation efficiency of siRNA, which revealed an encapsulation efficiency of 57 to 72% and a weight percent loading of 34 to 41% (*SI Appendix*, Table S1), corresponding to approximately 1,000 siRNA molecules per particle.

To validate the efficacy of LbL NPs in vivo, we first assessed the biodistribution of Cy7-labeled siSE-LbL NPs after both intraperitoneal (i.p.) and intravenous (i.v.) injection in the orthotopic model of ovarian cancer 24 h after administration (Fig. 5*D*). Consistent with previous observations (37), we observed more accumulation of LbL NPs into the intraperitoneal tumor nodules for i.p. administered NPs relative to i.v. administered NPs, based on near-infrared fluorescent tissue scanning and the quantification of percentage fluorescence intensity in tumor (*SI Appendix*, Fig. S7*B*). We then i.p. administered the siSE-LbL and siNT-LbL NPs to the orthotopic tumor-bearing animals (Fig. 5*E*) and observed 67% knockdown in SMARCE1 mRNA levels after four sequential injections, one dose per week (Fig. 5*F*). Urine samples from each treated animal were collected between 1 to 2 h after the last particle injection based on previous mathematical modeling (38). We detected significantly decreased urinary reporter concentration in the urine of tumor-bearing mice treated with siSE-LbL NPs relative to mice that received siNT-LbL NPs, signaling that the observed SMARCE1 knockdown was paralleled by decreased MMP protease activity (Fig. 5*G*). Notably, additional ECM invasion-related proteases, including MMP2, 9, 13 and PLAU, were also reduced, to varying degrees, in siSE-LbL-treated tumor nodules (*SI Appendix*, Fig. S7*C*). These results demonstrate that the single-particle siSE-LbL formulation was able to silence SMARCE1 activity in vivo, and that this silencing was paired with functional reduction in prometastatic gene expression patterns that could be monitored by a noninvasive urinary reporter.

## Discussion

Here, we establish a self-reporting RNAi delivery system for simultaneous knockdown of a transcription factor that drives disease progression and noninvasive monitoring of the resultant cell state. Specifically, we utilized a theranostic LbL NP loaded with a high density of siRNA that targets the proinvasive transcription factor SMARCE1, and also surface-functionalized with a biosensing peptide to detect downstream MMP protease activity that serves as a surrogate knockdown readout. We demonstrate that SMARCE1 is a relevant siRNA target for early-stage ovarian cancer. We show that inducible knockdown of SMARCE1 decreases metastatic potential in vitro and in vivo. Reduction of SMARCE1 also alters the expression and activity of a number of proteases that we detected by mRNA expression, protein abundance, and FRET-based peptide cleavage assays. Finally, we demonstrate that LbL NPs can quantify protease activity in response to reduction of SMARCE1 in vivo, thus resulting in a pharmacodynamic reporter of siRNA delivery and efficacy in ovarian cancer. Recent studies have determined ovarian cancer originates in the fallopian tube as serous tubal intraepithelial carcinoma (STIC) lesions and can be present for 7 to 8 y before spreading to the ovary (39, 40). With this knowledge in mind, targeting spread and invasion in ovarian cancer may be essential in treating the disease in its early stages (41), and it will be informative and important to explore how the siSE-LbL NPs perform in other animal models of ovarian cancer, particularly those that recapitulate early disease (42).

The modular nature of the single-particle LbL NP approach allows for the platform to be applied to many other targets that also benefit from paired downstream activity sensing. Future studies could involve similar knockdown analysis of other cancer-associated genes and transcription factors, such as oncogenes (KRASG12D, EGFR, and VEGF), proinflammatory transcription factors (NF-kB, AP-1, and STAT3), and cell proliferation transcription factors (c-MYC, E2F, and ETS1), followed by downstream protease activity analysis (43). We have developed activity-based sensor technologies that allow us to identify downstream proteases and inhibitors of virtually any target (44). In our current study, we chose a broad spectrum MMP substrate, but future work can employ alternate, more narrowly susceptible substrates to look more directly at how knockdown of a given target influences particular cancer hallmarks, such as angiogenesis (MMP1,2,9; KLK 2,6,7), inflammation (MMP8, ADAM17, and Legumain), or immune evasion (MMP8,12; DPP4). Additionally, the updated chemistry incorporated into the current LbL theranostic nanoparticles allows for the attachment of multiplexed reporters that could allow the simultaneous analysis of many proteases using a single particle formulation. Multiplexing reporters could also aid in discriminating reporter signal when a patient has other underlying diseases (such as arthritis, inflammation) that result in increased MMP activity (45, 46). Similar click chemistry reactions can be utilized to decorate the nanoparticles with homing peptides, such as iRGD, could also improve the specificity of delivery, enhance uptake, and reduce off-target toxicity (35, 36). We have also recently shown that LbL NPs can selectively target ovarian tumors through careful selection of the outermost polyelectrolyte layer (37). In particular, carboxylated polypeptide surface chemistries have enabled the development of effective combination therapy (47) and immunotherapy (48, 49) delivery platforms.

Despite the clear advantages and benefits of the LbL NP self-reporting theranostics, one limitation of our work is that we have yet to achieve the same level of SMARCE1 knockdown with nanoparticle treatment relative to what is observed in the continuous knockdown model. Future work will involve further optimization of dosing regimens to achieve maximal knockdown and decrease tumor dissemination. We did note that i.p. administration delivered more nanoparticles to the ovarian nodules than i.v. for this model. Additionally, previous studies have suggested knockdown of SMARCE1 sensitizes ovarian cancer to chemotherapy treatment, and thus testing a combination treatment with carboplatin or paclitaxel, the current regimen of ovarian cancer chemotherapy treatment, may optimize tumor regression (50). In either case, having the urinary biomarker to quickly and accessibly report on the functional impact of SMARCE1 delivery remains a strong advantage of the LbL NP platform.

Nucleic acid therapeutics have enormous potential to interfere with cancer progression and metastasis. Combining these therapeutics with protease activity sensing enables us to develop theranostics that yield detailed information regarding the efficacy of both delivery and downstream outcome of a therapeutic. This activity-based pairing allows for earlier determination of treatment efficacy to enable fine-tuned dosing regimens or informative clues as to when to pivot to other therapies. Additionally, future studies will involve modifying these nanoparticles to knockdown therapeutic targets in other cancer types, as well as other disease indications. We envision this work can directly benefit siRNA therapies in current clinical development that target clotting factors to treat thrombosis and hemophilia, as these targets are directly upstream of the protease thrombin (51, 52). We have performed extensive work to explore the rich protease biology underpinning a number of diseases such as lung pathologies (53–55), pneumonia (56), and bacterial infection (57), which could be leveraged to enhance RNAi delivery in these indications. In all, we report the development of a modular, self-reporting RNAi nanoparticle to enable therapeutic interference and monitoring of disease progression.

## Materials and Methods

### Reagents.

1,2-distearoyl-sn-glycero-3-phospho-(1′-rac-glycerol) (sodium salt) (DSPG), 1,2-dioleoyl-sn-glycero-3-phosphoethanolamine (DOPE), and cholesterol were purchased from Avanti. Sulfo-cyanine 7 NHS ester was purchased from Lumiprobe. Chloroform and methanol were purchased from TCI and Sigma, respectively. Whatman nuclepore polycarbonate hydrophilic membranes (400, 200, 100, and 50 nm sizes) were purchased from GE. All glassware was obtained from Chemglass. 50/15 mL Falcon tubes and 50/5/2 mL DNA loBind Eppendorf tubes were purchased from VWR. Locked nucleic acid siRNAs (Silencer Select), 5 M bioreagent grade NaCl solution, and 1 M bioreagent-grade HEPES were purchased from Fisher Scientific. The siRNA sequences (sense) used were CCC AUA CCA GAA GAU GAG AAA (siSMARCE1) and UUC UCC GAA CGU GUC ACG U (scramble control, *SI Appendix*, Table S4). Both sequences were purchased from the “in vivo ready” Thermo Fisher platform, containing proprietary chemical modifications suitable for in vivo knockdown. D02-E100-05-N and C02-E100-05-N tangential flow filtration filters were purchased from Spectrum Labs (now Repligen). Poly-L-arginine hydrochloride (38.5 kDa) was purchased from Alamanda Polymers. Propargyl modified poly-L-aspartic acid was synthesized and characterized as described previously (28). Polystyrene semi-micro cuvettes for the Malvern Zetasizer were purchased from VWR, and DTS1070 folded capillary cells were purchased directly from Malvern. Recombinant MMP9 was purchased from Enzo Life Sciences. The azido-modified biosensor peptide (sequence B(biotin)-eGvndneeGffsarK-(FAM) dGGPLGVRGKK-(N3)) was synthesized by CPC Scientific with >90% purity. mPEG-azide (5 kDa) was purchased from Creative PEGWorks.

### Preparation of Liposomes.

Liposomes were prepared as previously described (28). Cholesterol and DOPE were dissolved in chloroform, and DSPG was dissolved in a 65:35:8 volume ratio of chloroform, methanol, and deionized water (milli-Q). These combinations of lipids were chosen based on previous literature about optimal liposome composition for in vivo drug delivery (58). Lipid mixtures composed of 66.7 mol% DSPG and 33.3 mol% cholesterol were prepared in round bottom flasks (RBFs) (10 or 50 mL depending on scale), and for DSPG-formulations, methanol was added dropwise until the solution cleared. For fluorescent liposomes, the formulation was 61.7 mol% DSPG, 5 mol% DOPE and 33.3 mol% cholesterol. The lipid solution was evaporated using a BUCHI RotoVap system under heat (60 °C, water bath) until completely dry (<15 mBarr). A Branson sonicator bath was filled with reverse-osmosis water and heated until >70 °C, at which point the RBF with the lipid film was partially submerged in the bath and a volume of dH2O was added to resuspend the lipid film to a 1 mg lipid/mL solution. The liposome solution was sonicated for roughly 1 min and then transferred to an Avestin LiposoFast LF-50 liposome extruder. The extruder was connected to a Cole-Parmer Polystat Heated Recirculator Bath to maintain a temperature >65 °C. The liposome solution was extruded through successively smaller nuclepore membranes until a 50-100-nm liposome was obtained. This requires 1 pass through a stack of 2-3 400, 200, 100, and 50 nm membranes. Fluorescently labeled liposomes were prepared via standard NHS-coupling of Sulfo-cyanine NHS ester to DOPE head groups according to manufacturer instructions. Liposomes were characterized for size and zeta using the techniques outlined below.

### Preparation of Layer-by-layer Nanoparticles.

Layering was carried out as described previously (28). Nanoparticles were layered by adding an equal volume of nanoparticle solution (not exceeding 1 mg core template/mL) to an equal volume of polyelectrolyte solution under sonication (Branson bath sonicator, room temperature). The mixture was sonicated for roughly 5 s. The weight equivalents (wt. eq.) of polyelectrolyte used with respect to the liposome core were 0.5 (siRNA), 0.33 (PLR), and 0.3 (pPLD). Polyelectrolyte solutions were always prepared in 50 mM HEPES and 40 mM NaCl (pH 7.4), which, upon mixing with the NP substrate (in water), were diluted 1:1 to 25 mM HEPES and 20 mM NaCl final concentration. DNA loBind tubes were used as the mixing vessels for all experiments to prevent nonspecific adsorption of siRNA and other polymers to the plastic.

The layered particle was allowed to incubate at room temperature for 1 h and was then purified using the tangential flow filtration method, as described previously (28). Briefly, crude nanoparticle solution is connected to a Spectrum Labs KrosFlo II system using masterflex, Teflon-coated tubing. D02-E100-05-N (batch volume > 5 mL) or C02-E100-05-N (batch volume < 1 mL) 100 kDa filters were used to purify the particles until 5 volume-equivalents were collected in the permeate. For cationic layers, the TFF filter was pretreated with a solution of free polycation (same concentration used for layering) in order to minimize nonspecific adsorption of particles to the membrane walls. Samples were run at flow rates of 80 mL/min (size 16 tubing, used with D02-series filters) or 13 mL/min (size 13 tubing, used with C02-series filters). Once pure, the sample was either concentrated (by disconnecting the buffer reservoir) or recovered via reversing the direction of the peristaltic pump. For purification, the exchange buffer was always Milli-Q water. For more complete yields, 1-3 mL of water was run backward through the tubing to recover any remaining particles. This process was repeated until the desired LbL formulation was obtained.

### Click-chemistry Conjugation onto LbL Nanoparticles.

This procedure was carried out as described previously (28). After formulation, LbL NPs were modified with azide functionalized ligands by mixing copper II sulfate, tris-hydroxypropyltriazolylmethylamine (THPTA), and sodium ascorbate. All solutions with the exception of azide-functionalized ligand were prepared in water. The azide functionalized ligand was dissolved in 0.1× PBS, though HEPES and water have also worked equally well in a representative example, 0.05 mL of a 1 mg/mL LbL NP solution was mixed with 0.025 mL of azide-functionalized biosensor peptide (0.28 to 1.12 mM), 0.0875 mL sodium ascorbate (1 mg/mL), and 0.0875 mL CuSO4/THPTA solution (0.016 mg/mL CuSO4, 0.0672 mg/mL THPTA). This reaction was scaled as needed. All reactions were carried out at room temperature protected from light over 12 to 16 h. Subsequently, tangential flow filtration was used to remove excess reagents and concentrate functionalized LbL NPs to the desired concentration for experiments. The fluorescence was measured as a proxy of peptide conjugation efficiency and used to ensure similar conjugation efficiency of peptide between siNT and siSE particles (*SI Appendix*, Table S1).

### Characterization of Nanoparticles.

Nanoparticle hydrodynamic size and polydispersity were measured using dynamic light scattering (Malvern ZS90 Particle Analyzer, λ = 633 nm, material/dispersant RI 1.590/1.330). Zeta potential measurements were also acquired with the Malvern ZS90, using laser Doppler electrophoresis. Nanoparticle solutions were diluted in milli-Q water in polystyrene semi-micro cuvettes (VWR) or DTS1070 folded capillary cuvettes (Malvern) to produce samples for characterization on the Malvern SZ90. siRNA content was measured as described previously (28). Encapsulation efficiency (EE%) was calculated as EE% = [siRNA (actual))/(siRNA (theoretical)] × 100. For the particles utilized in these studies, the EE were determined to be 57 and 72% for siSMARCE1 and scramble siRNA containing LbL NPs, respectively. This corresponds to weight % loading values of 34 and 43%.

#### Survival curve analysis.

Ovarian cancer patient data were publicly available in the Kaplan–Meier Plotter database (http://kmplot.com/analysis/index.php?p=service&cancer=ovar) and were analyzed for overall survival. Survival curves in ovarian cancer patients stratified into tertiles were plotted based on tumor SMARCE1 expression in cohorts of patients with early-stage ovarian tumors (stage I or II; n = 179) or late-stage ovarian tumors (stages III and IV; n = 1,268).

#### Cell culture.

Human ovarian cancer cell line OVCAR-8-LucF with doxycycline (DOX)-inducible SMARCE1 shRNA (from National Cancer Institute 60 Human Tumor Cell Lines Screen) was cultured in RPMI (Gibco) medium supplemented with 10% (v/v) fetal bovine serum (FBS) (Gibco), 1% (v/v) penicillin/streptomycin (CellGro), and puromycin (2 µg/mL) at 37 °C and in 5% CO_2_. Cell lines tested negative for *Mycoplasma* contamination.

#### Animal models.

All animal studies were approved by the Massachusetts Institute of Technology (MIT) committee on animal care (MIT protocol 0417-025-20 & 0217-014-20). All experiments were conducted in compliance with institutional and national guidelines and supervised by the Division of Comparative Medicine (DCM) of MIT staff. To make the ovarian cancer model, NCr nude female mice (4 to 5 wk of age) were inoculated by intraperitoneal (IP) (orthotopic model) or intravenous (IV) (metastatic model) injection with human cell lines (2 M cells/mouse for IP, 1 M cells/mouse for IV, and OVCAR-8-LucF) expressing firefly luciferase. Mice were then put on a special diet of doxycycline (200 mg/kg). Tumor progression was monitored weekly using IVIS Imaging Systems (IVIS, PerkinElmer). By the end time point of each study, mice were killed, and tumor tissue was collected for further analysis.

#### Analysis of urine from fluorescent particle injections.

Nanoparticles (200 µL) or theranostic particles (200 µL) were injected into experimental mice via IP (orthotopic model) or IV (metastatic model) injection. Mice were then placed into custom housing with a 96-well plate base for urine collection. The bladders were voided to collect between 100 and 200 µL of urine after 2 h (if IP) or 1 h (if IV). Cleaved substrate concentration in urine was quantified using ELISA according to the manufacturer’s protocol (R&D Systems).

#### Histology and immunohistochemistry (IHC) studies.

Paraffin-embedded tissues were preserved in 4% paraformaldehyde (PFA) overnight and stored in 70% ethanol prior to embedding into paraffin. Snap-frozen tissues were preserved in 2% PFA for 2 h, stored in 30% sucrose overnight, and frozen in optimum cutting temperature (OCT) compound at −80 °C. Samples were sectioned into 6-µm slices and stained for further analysis. For IHC studies, slides were stained with primary antibodies in accordance with manufacturer instructions, followed by HRP secondaries. Slides were sealed with ProLong Antifade Mountants (Thermo Scientific). The ovarian cancer and normal tissue microarrays were obtained from US Biomax (catalog number BC11012). Blind expression scoring of cores was performed by R. T. Bronson at the KI Histology Core. Slides were digitized and analyzed using an 3D Histech P250 High Capacity Slide Scanner (Perkin Elmer). Antibodies and dilutions used were listed in *SI Appendix*, Table S3.

#### RNA extraction and RT-qPCR.

Tissues samples were kept in RNAlater RNA Stabilization Reagent (Qiagen, Inc.). RNA from tissue after cryoground was extracted using RNeasy Mini Kit (Qiagen, Inc.). RNA was reverse transcribed into cDNA using BioRad iScript Reverse Transcription Supermix. qPCR amplification of the cDNA was measured after mixing with Taqman gene expression probes and Applied Biosystems TaqMan Fast Advanced Master Mix (Thermo Scientific) according to the manufacturer’s instructions. To probe multiplexed tumor invasion related genes, TaqMan™ Array Human Extracellular Matrix & Adhesion Molecules (Thermo Fisher) was used according to instructions from manufacturer’s kit.

#### Protein extraction and tissue lysate proteolytic cleavage assay.

Tissue samples were homogenized in PBS and centrifuged at 4 °C for 5 min at 6,000 × g. The supernatant was further centrifuged at 14,000 × g for 25 min at 4 °C. Protein concentration was measured using the ThermoFisher BCA Protein Assay Kit and prepared at 2 mg/mL prior to assay. Assays were performed in the 384-well plate in triplicate in enzyme-specific buffer with peptides (1 µM) and cell lysates (0.33 mg/mL) in 30 µL at 37 °C. Fluorescence was measured at Ex/Em 495/535nm using a Tecan Infinite 200pro microplate reader (Tecan). Signal increase at 60 min was used across conditions.

#### Protease array.

The Proteome Profiler Human Protease Array Kit (R&D) was used according to the manufacturer’s instructions. The Frozen Tissue Array (Biochain) was used to analyze 37 different ovarian tumors and 3 corresponding normal controls (5 slides).

#### Matrigel invasion assay.

Human cell line OVCAR-8 was treated with doxycycline (2 mg/mL in PBS) such that the final concentration was 5 µg/mL and incubated overnight at 37 °C. The following day, matrigel (0.1 mg/mL in RPMI, Corning) was plated onto 12 transwells (24-well plate, Corning) and incubated at 37 °C for 1 h or until matrigel polymerized. OVCAR-8 cells were trypsinized, counted, and centrifuged for 5 min at 1,000 rpm. Media were aspirated from the pellet, and cells were resuspended in low-serum media (1% v/v FBS in RPMI) such that cell concentration was 200 K cells/2 mL. Cell suspension (200 µL) was added to transwells, and high-serum media (500 µL, 10% FBS in RPMI) was added to the outer membrane compartment of transwells. Cells were incubated at 37 °C for 20 h. Media were then aspirated from transwells, and cells were washed with PBS and fixed with 4% PFA for 10 min. Cells were then washed 3 times with PBS. DAPI (0.5 µg/mL in PBS, 2 mL) was added to transwells for 30 min. Cells were washed three times with PBS and left in PBS (2 mL). Photos of membrane were taken (four photos per well). Matrigel was carefully removed by swabbing transwell membranes three times per well. Cells were left in PBS (2 mL) and imaged again (four photos per well). The number of cells per photo was quantified in ImageJ. The average number of cells per photo was used to calculate the number of cells per well using the following formula: n_well_ = (n_photo_1 + n_photo_2 + n_photo_3 + n_photo_4)/4 × Area_well_ /Area_photo_

### Statistical Analysis.

Statistical analyses were conducted in GraphPad Prism (v. 8.4.1). Data are presented as means with SEM. To determine whether there are any statistically significant differences between the means of two or more independent groups, the Student’s *t* test was used for paired data, and the one-way ANOVA was used when there was a minimum of three groups. Sample sizes and the statistical test are specified in the figure legends.

## Supplementary Material

Appendix 01 (PDF)

Dataset S01 (XLSX)

Dataset S02 (XLSX)

## Data Availability

All study data are included in the article and/or supporting information.

## References

[1] T. R. Damase , The limitless future of RNA therapeutics. Front. Bioeng. Biotechnol. **9**, 628137 (2021). 33816449 10.3389/fbioe.2021.628137 PMC8012680

[2] S. A. Lim, A. Cox, M. Tung, E. J. Chung, Clinical progress of nanomedicine-based RNA therapies. Bioact. Mater. **12**, 203–213 (2021). 35310381 10.1016/j.bioactmat.2021.10.018 PMC8897211

[3] M. May Zhang, R. Bahal, T. P. Rasmussen, J. E. Manautou, X. Zhong, The growth of siRNA-based therapeutics: Updated clinical studies. Biochem. Pharmacol. **189**, 114432 (2021). 33513339 10.1016/j.bcp.2021.114432 PMC8187268

[4] G. M. Traber, A.-M. Yu, RNAi-based therapeutics and novel RNA bioengineering technologies. J. Pharmacol. Exp. Ther. **384**, 133–154 (2023). 35680378 10.1124/jpet.122.001234 PMC9827509

[5] Z. Tao, X. Wu, Targeting transcription factors in cancer: From “undruggable” to “druggable”. Methods Mol. Biol. **2594**, 107–131 (2023). 36264492 10.1007/978-1-0716-2815-7_9 PMC9724592

[6] Y.-K. Kim, RNA therapy: Rich history, various applications and unlimited future prospects. Exp. Mol. Med. **54**, 455–465 (2022). 35440755 10.1038/s12276-022-00757-5 PMC9016686

[7] S. D. Solomon , Effects of patisiran, an RNA interference therapeutic, on cardiac parameters in patients with hereditary transthyretin-mediated amyloidosis. Circulation **139**, 431–443 (2019). 30586695 10.1161/CIRCULATIONAHA.118.035831 PMC12611557

[8] Y. Qiu, J. K. W. Lam, S. W. S. Leung, W. Liang, Delivery of RNAi therapeutics to the airways—From Bench to Bedside. Molecules **21**, 1249 (2016). 27657028 10.3390/molecules21091249 PMC6272875

[9] D. Adams , Patisiran, an RNAi therapeutic, for hereditary transthyretin amyloidosis. N Engl. J. Med. **379**, 11–21 (2018). 29972753 10.1056/NEJMoa1716153

[10] E. Sardh, P. Harper, RNAi therapy with givosiran significantly reduces attack rates in acute intermittent porphyria. J. Int. Med. **291**, 593–610 (2022). 10.1111/joim.13443 35067977

[11] Y. Frishberg , Phase 1/2 study of lumasiran for treatment of primary hyperoxaluria type 1: A placebo-controlled randomized clinical trial. Clin. J. Am. Soc. Nephrol. **16**, 1025 (2021). 33985991 10.2215/CJN.14730920 PMC8425611

[12] G. Santulli, S. S. Jankauskas, J. Gambardella, Inclisiran: A new milestone on the PCSK9 road to tackle cardiovascular risk. Eur. Heart J. Cardiovasc. Pharmacother. **7**, e11–e12 (2021). 33655296 10.1093/ehjcvp/pvab014 PMC13376130

[13] T. Golan , RNAi therapy targeting KRAS in combination with chemotherapy for locally advanced pancreatic cancer patients. Oncotarget **6**, 24560–24570 (2015). 26009994 10.18632/oncotarget.4183 PMC4695206

[14] B. Schultheis , Safety, efficacy and pharcacokinetics of targeted therapy with the liposomal RNA interference therapeutic Atu027 combined with gemcitabine in patients with pancreatic adenocarcinoma. A randomized phase Ib/IIa study. Cancers (Basel) **12**, 3130 (2020). 33114652 10.3390/cancers12113130 PMC7693593

[15] Moderna Inc., Moderna and Merck Announce mRNA-4157 (V940) in Combination With KEYTRUDA(R) (pembrolizumab) Demonstrated a Statistically Significant and Clinically Meaningful Improvement in Distant Metastasis-Free Survival in Patients with High-Risk Stage III/IV Melanoma Following Complete Resection Versus KEYTRUDA. https://investors.modernatx.com/news/news-details/2023/Moderna-and-Merck-Announce-mRNA-4157-V940-in-Combination-With-KEYTRUDAR-pembrolizumab-Demonstrated-a-Statistically-Significant-and-Clinically-Meaningful-Improvement-in-Distant-Metastasis-Free-Survival-in-Patients-with-High-Risk-Stage-IIIIV-Melanoma-F/default.aspx. Accessed 6 June 2023.

[16] E. Sanz-Garcia, E. Zhao, S. V. Bratman, L. L. Siu, Monitoring and adapting cancer treatment using circulating tumor DNA kinetics: Current research, opportunities, and challenges. Sci. Adv. **8**, eabi8618 (2022). 35080978 10.1126/sciadv.abi8618 PMC8791609

[17] J. Ronald, H. Chuang, A. Dragulescu-Andrasi, S. Hori, S. Gambhir, Detecting cancers through tumor-activatable minicircles that lead to a detectable blood biomarker. Proc. Natl. Acad. Sci. U.S.A. **112**, 3068–3073 (2015). 25713388 10.1073/pnas.1414156112 PMC4364239

[18] E. R. Robinson , Minicircles for a two-step blood biomarker and PET imaging early cancer detection strategy. J. Control Release **335**, 281–289 (2021). 34029631 10.1016/j.jconrel.2021.05.026 PMC8262353

[19] A. Akinc , A combinatorial library of lipid-like materials for delivery of RNAi therapeutics. Nat. Biotechnol. **26**, 561–569 (2008). 18438401 10.1038/nbt1402 PMC3014085

[20] S. C. Semple , Rational design of cationic lipids for siRNA delivery. Nat. Biotechnol. **28**, 172–176 (2010). 20081866 10.1038/nbt.1602

[21] K. A. Whitehead , Degradable lipid nanoparticles with predictable in vivo siRNA delivery activity. Nat. Commun. **5**, 4277 (2014). 24969323 10.1038/ncomms5277 PMC4111939

[22] A. Akinc , The Onpattro story and the clinical translation of nanomedicines containing nucleic acid-based drugs. Nat. Nanotechnol. **14**, 1084–1087 (2019). 31802031 10.1038/s41565-019-0591-y

[23] A. P. Soleimany, S. N. Bhatia, Activity-based diagnostics: An emerging paradigm for disease detection and monitoring. Trends Mol. Med. **26**, 450–468 (2020). 32359477 10.1016/j.molmed.2020.01.013 PMC8290463

[24] E. J. Kwon, J. S. Dudani, S. N. Bhatia, Ultrasensitive tumour-penetrating nanosensors of protease activity. Nat. Biomed. Eng. **1**, 0054 (2017). 28970963 10.1038/s41551-017-0054 PMC5621765

[25] Z. J. Deng , Layer-by-layer nanoparticles for systemic codelivery of an anticancer drug and siRNA for potential triple-negative breast cancer treatment. ACS Nano **7**, 9571–9584 (2013). 24144228 10.1021/nn4047925 PMC3870477

[26] J. J. Chou, A. G. Berger, S. Jalili-Firoozinezhad, P. T. Hammond, A design approach for layer-by-layer surface-mediated siRNA delivery. Acta Biomaterialia **135**, 331–341 (2021). 34481054 10.1016/j.actbio.2021.08.042 PMC9316412

[27] N. Boehnke, K. J. Dolph, V. M. Juarez, J. M. Lanoha, P. T. Hammond, Electrostatic conjugation of nanoparticle surfaces with functional peptide motifs. Bioconjug. Chem. **31**, 2211–2219 (2020). 32786506 10.1021/acs.bioconjchem.0c00384 PMC7895459

[28] N. Boehnke , Theranostic layer-by-layer nanoparticles for simultaneous tumor detection and gene silencing. Angew. Chem. Int. Ed. Engl. **59**, 2776–2783 (2020). 31747099 10.1002/anie.201911762 PMC7002217

[29] American Society of Clinical Oncology, Ovarian, Fallopian Tube, and Peritoneal Cancer–Statistics. Cancer. Net (2012). https://www.cancer.net/cancer-types/ovarian-fallopian-tube-and-peritoneal-cancer/statistics. Accessed 6 June 2023.

[30] E. S. Sokol , SMARCE1 is required for the invasive progression of in situ cancers. Proc. Natl. Acad. Sci. U.S.A. **114**, 4153–4158 (2017). 28377514 10.1073/pnas.1703931114 PMC5402464

[31] H. Lomelí, J. Castillo-Robles, The developmental and pathogenic roles of BAF57, a special subunit of the BAF chromatin-remodeling complex. FEBS Lett. **590**, 1555–1569 (2016). 27149204 10.1002/1873-3468.12201

[32] N. Hah , A role for BAF57 in cell cycle-dependent transcriptional regulation by the SWI/SNF chromatin remodeling complex. Cancer Res. **70**, 4402–4411 (2010). 20460533 10.1158/0008-5472.CAN-09-2767 PMC2880201

[33] H. Suzumura , BAF57 is a potential determinant of colorectal cancer malignancy. Anticancer Res. **41**, 5445–5452 (2021). 34732413 10.21873/anticanres.15356

[34] J. S. Appelbaum , Arginine topology controls escape of minimally cationic proteins from early endosomes to the cytoplasm. Chem. Biol. **19**, 819–830 (2012). 22840770 10.1016/j.chembiol.2012.05.022 PMC3488872

[35] J. H. Lo , iRGD-guided tumor penetrating nanocomplexes for therapeutic siRNA delivery to pancreatic cancer. Mol. Cancer Ther. **17**, 2377–2388 (2018). 30097486 10.1158/1535-7163.MCT-17-1090 PMC6298224

[36] K. N. Sugahara , Tissue-penetrating delivery of compounds and nanoparticles into tumors. Cancer Cell **16**, 510–520 (2009). 19962669 10.1016/j.ccr.2009.10.013 PMC2791543

[37] S. Correa , Tuning nanoparticle interactions with ovarian cancer through layer-by-layer modification of surface chemistry. ACS Nano **14**, 2224–2237 (2020). 31971772 10.1021/acsnano.9b09213 PMC7062411

[38] G. A. Kwong , Mathematical framework for activity-based cancer biomarkers. Proc. Natl. Acad. Sci. U.S.A. **112**, 12627–12632 (2015). 26417077 10.1073/pnas.1506925112 PMC4611621

[39] S. I. Labidi-Galy , High grade serous ovarian carcinomas originate in the fallopian tube. Nat. Commun. **8**, 1093 (2017). 29061967 10.1038/s41467-017-00962-1 PMC5653668

[40] R. Perets, R. Drapkin, It’s totally tubular…riding the new wave of ovarian cancer research. Cancer Res. **76**, 10–17 (2016). 26669862 10.1158/0008-5472.CAN-15-1382 PMC4703449

[41] T. M. Bergsten, J. E. Burdette, M. Dean, Fallopian tube initiation of high grade serous ovarian cancer and ovarian metastasis: Mechanisms and therapeutic implications. Cancer Lett. **476**, 152–160 (2020). 32067992 10.1016/j.canlet.2020.02.017 PMC7069002

[42] S. Stuckelberger, R. Drapkin, Precious GEMMs: Emergence of faithful models for ovarian cancer research. J. Pathol. **245**, 129–131 (2018). 29493783 10.1002/path.5065

[43] K. Vishnoi, N. Viswakarma, A. Rana, B. Rana, Transcription factors in cancer development and therapy. Cancers (Basel) **12**, 2296 (2020). 32824207 10.3390/cancers12082296 PMC7464564

[44] A. P. Amini , Multiscale profiling of protease activity in cancer. Nat. Commun. **13**, 5745 (2022). 36192379 10.1038/s41467-022-32988-5 PMC9530178

[45] B. Grillet , Matrix metalloproteinases in arthritis: Towards precision medicine. Nat. Rev. Rheumatol. **19**, 363–377 (2023). 37161083 10.1038/s41584-023-00966-w

[46] S. Löffek, O. Schilling, C.-W. Franzke, Biological role of matrix metalloproteinases: A critical balance. Eur. Respiratory J. **38**, 191–208 (2011). 10.1183/09031936.00146510 21177845

[47] S. Kong , Synergistic combination therapy delivered via layer-by-layer nanoparticles induces solid tumor regression of ovarian cancer. Bioeng. Trans. Med. **8**, e10429 (2023). 10.1002/btm2.10429 PMC10013771 36925689

[48] A. E. Barberio , Cancer cell coating nanoparticles for optimal tumor-specific cytokine delivery. ACS Nano **14**, 11238–11253 (2020). 32692155 10.1021/acsnano.0c03109 PMC7530125

[49] A. E. Barberio , Layer-by-layer interleukin-12 nanoparticles drive a safe and effective response in ovarian tumors. Bioeng. Trans. Med. **8**, e10453 (2023). 10.1002/btm2.10453 PMC10013828 36925719

[50] T. Yamaguchi , Expression of BAF57 in ovarian cancer cells and drug sensitivity. Cancer Sci. **106**, 359–366 (2015). 25611552 10.1111/cas.12612 PMC4409878

[51] W. Lin , RNAi targeting heparin cofactor II promotes hemostasis in hemophilia A. Mol. Ther. Nucleic Acids **24**, 658–668 (2021). 33996250 10.1016/j.omtn.2021.03.022 PMC8093307

[52] M. Heestermans, B. J. M. van Vlijmen, Oligonucleotides targeting coagulation factor mRNAs: Use in thrombosis and hemophilia research and therapy. Thromb J. **15**, 7 (2017). 28286423 10.1186/s12959-017-0130-8 PMC5341404

[53] J. D. Kirkpatrick , Urinary detection of lung cancer in mice via noninvasive pulmonary protease profiling. Sci. Transl. Med. **12**, eaaw0262 (2020). 32238573 10.1126/scitranslmed.aaw0262 PMC7894603

[54] L. W. Chan , Engineering synthetic breath biomarkers for respiratory disease. Nat. Nanotechnol. **15**, 792–800 (2020). 32690884 10.1038/s41565-020-0723-4 PMC8173716

[55] A. P. Soleimany, C. Martin-Alonso, M. Anahtar, C. S. Wang, S. N. Bhatia, Protease activity analysis: A toolkit for analyzing enzyme activity data. ACS Omega **7**, 24292–24301 (2022). 35874224 10.1021/acsomega.2c01559 PMC9301967

[56] M. Anahtar , Host protease activity classifies pneumonia etiology. Proc. Natl. Acad. Sci. U.S.A. **119**, e2121778119 (2022). 35696579 10.1073/pnas.2121778119 PMC9231472

[57] C. Ngambenjawong, L. W. Chan, H. E. Fleming, S. N. Bhatia, Conditional antimicrobial peptide therapeutics. ACS Nano **16**, 15779–15791 (2022). 35980829 10.1021/acsnano.2c04162 PMC9619929

[58] S. M. Lee , A systematic study of unsaturation in lipid nanoparticles leads to improved mRNA transfection in vivo. Angew Chem. Int. Ed. Engl. **60**, 5848–5853 (2021). 33305471 10.1002/anie.202013927 PMC8100975

