# Supplementary material for: Targeting and monitoring ovarian cancer invasion with an RNAi and peptide delivery system
Source: Proc Natl Acad Sci U S A. 2024 Mar 4;121(11):e2307802121. doi: 10.1073/pnas.2307802121 (PMC10945808; doi:10.1073/pnas.2307802121)
Supplement: Supplementary file 1 — Appendix 01 (PDF) [file pnas.2307802121.sapp.pdf]

Supplementary Figures:

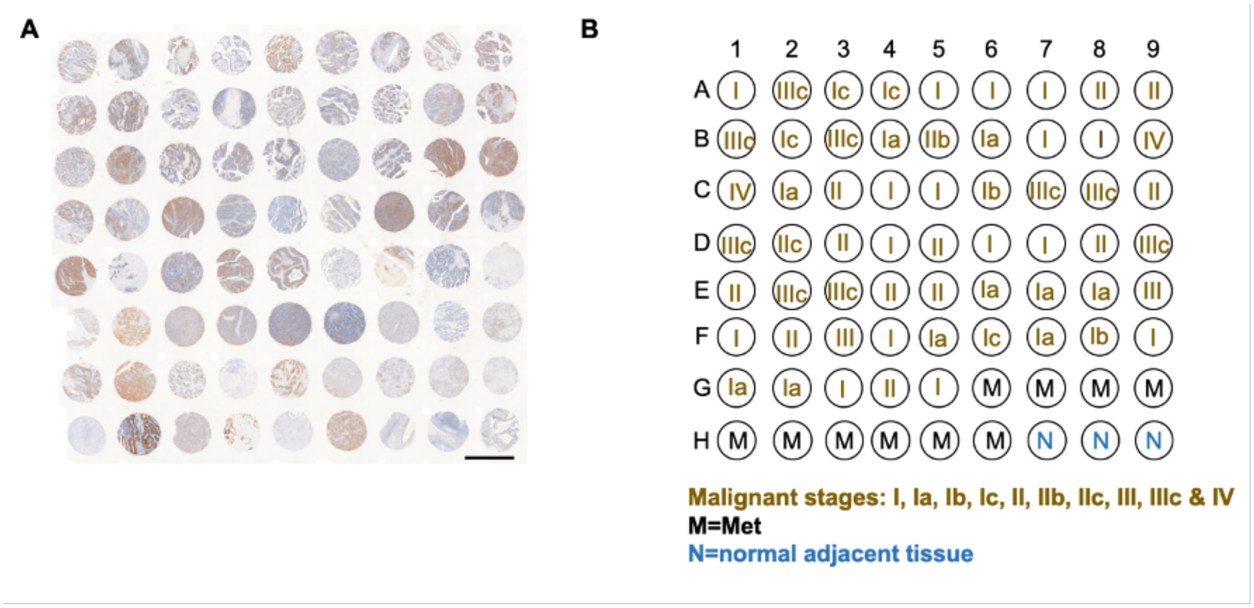

Fig S1. IHC staining of SMARCE1 in human Tissue Microarrays (TMAs). IHC staining of tumor samples at different malignant stages (I, Ia, Ib, Ic, II, IIb, IIc, III, IIIc & IVv I, Ia, Ib, Ic, II, IIb, IIc, III, IIIc & IV). Normal adjacent tissues were included as control samples.

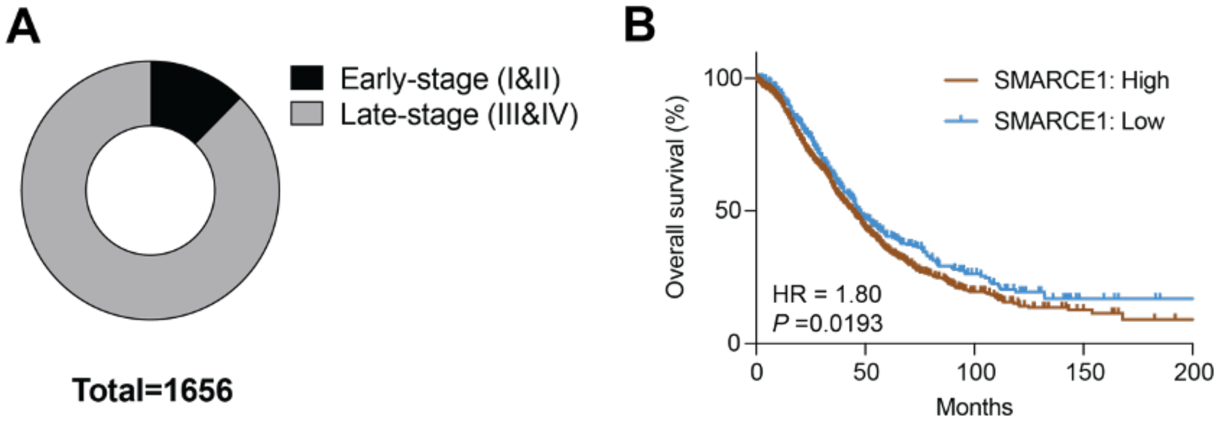

Fig S2. Ovarian cancer patient overall survival analysis. (**A**) Number of patients with available clinical data of tumor staging information. (**B**) Survival curves in ovarian cancer patients (Kaplan-Meier plotter, ovarian cancer) stratified into tertiles (high, low) were plotted based on tumor SMARCE1 expression. Hazard ratios (HRs) and *P* values were determined with the log-rank statistical test.

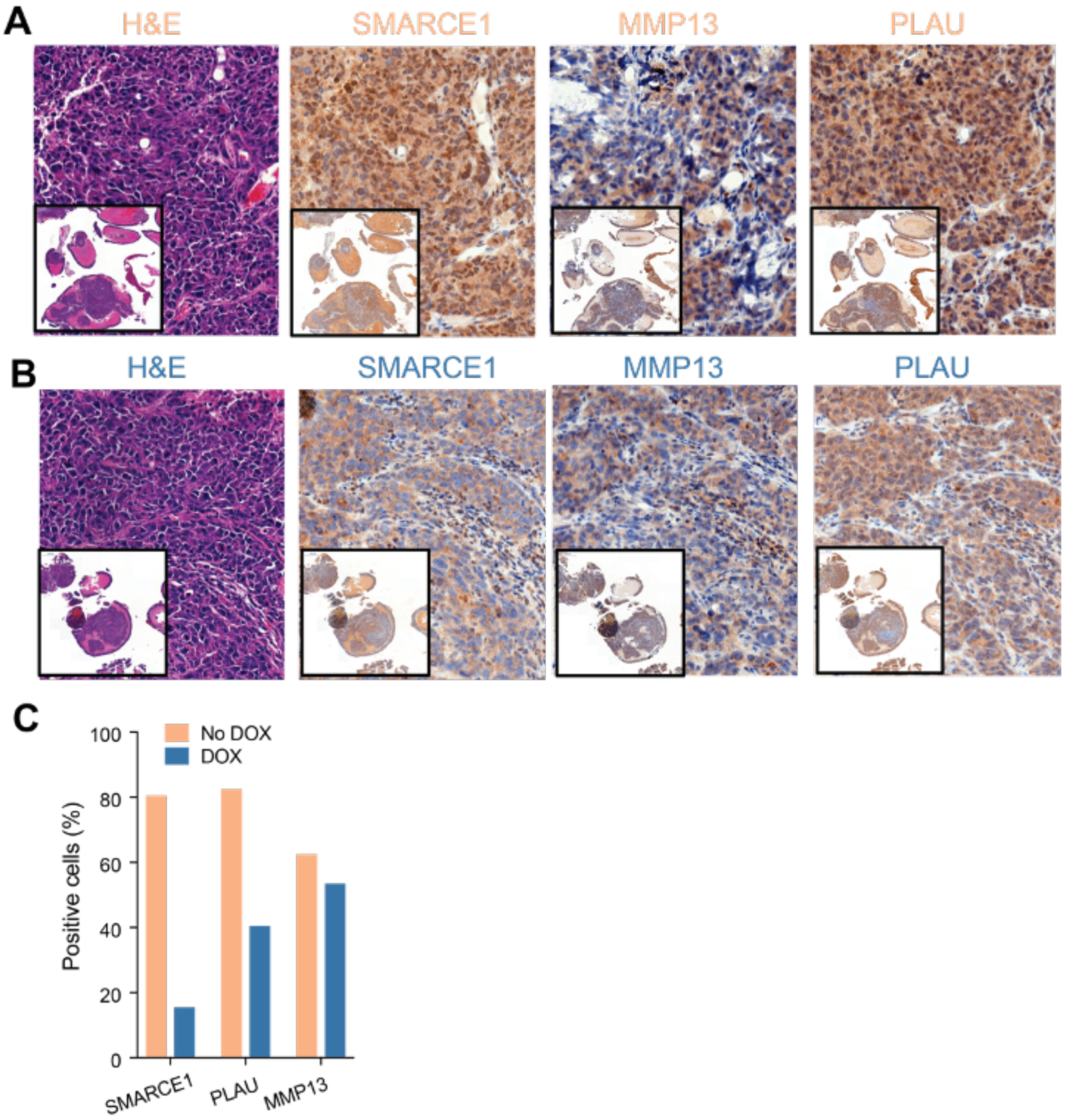

Fig S3. SMARCE1 knockdown inhibited ECM proteases expression. (**A**) Inducible orthotopic ovarian cancer tumors were implanted by i.p. injection of human OVCAR8 cell lines in female nude mice. Tumors without or with doxycycline treatment (**B**) were collected, sectioned, and stained with SMARCE1, MMP13 and PLAU antibodies in IHC. (**C**) Cells with positive staining in IHC were counted with a publicly available web application (www/immunoratio/).

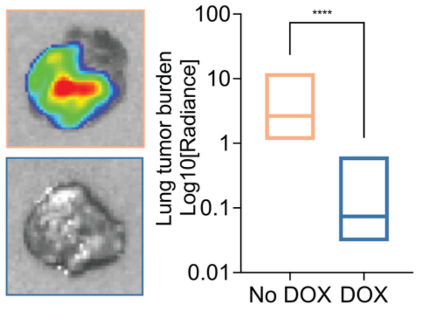

Fig S4. SMARCE1 knockdown induces reduced tumor burden in lung. Representative luminescence images and quantification of metastatic burden in whole lungs of mice intravenously inoculated with OVCAR8-shSMARCE1 fed with or without DOX chow diet for four weeks (mean ± s.e.m, n = 6 per group for animals on the DOX diet, n=3 for control group of animals on a standard diet, two-tailed Student's *t*-test, ****$P < 0.0001$).

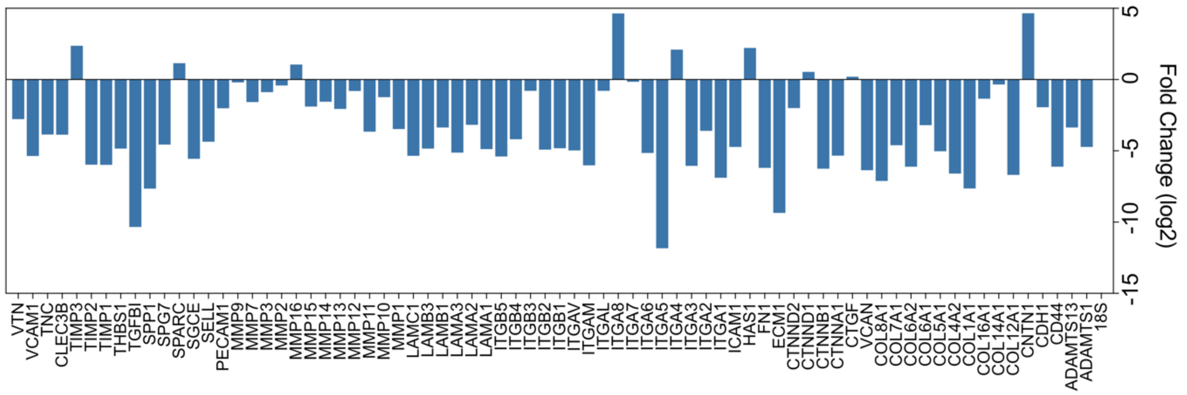

Fig S5. Quantification of change in protease transcripts after SMARCE1-shRNA knockdown in OVCAR8 tumor nodules. TaqMan™ Array Human Tumor Metastasis analysis quantifying mRNA transcripts from tumor nodules extracted from animals injected with OVCAR8-shSMARCE1 and fed with or without DOX chow diet. Gene expression is normalized to 18S RNA control and plotted as fold change relative to the control animals (n = 5 animals per group).

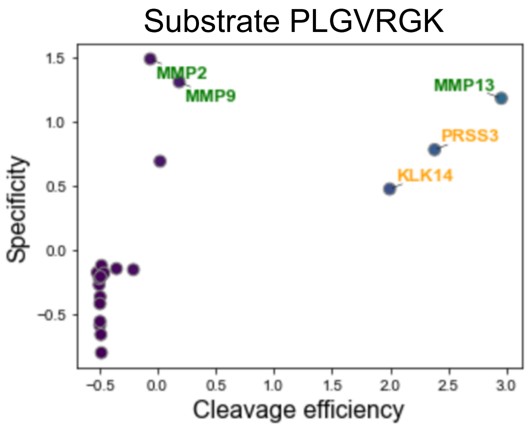

Fig S6. Identification of suspected proteases that cleave PLGVRGK. Previously published *in vitro* screening data of recombinant proteases that cleave the chosen urinary reporter sequence[57]. Cleavage efficiency is determined by the cleavage rate of the proteases. Specificity is determined by how well the given proteases cleaved the sequence compared to other sequences in the screen.

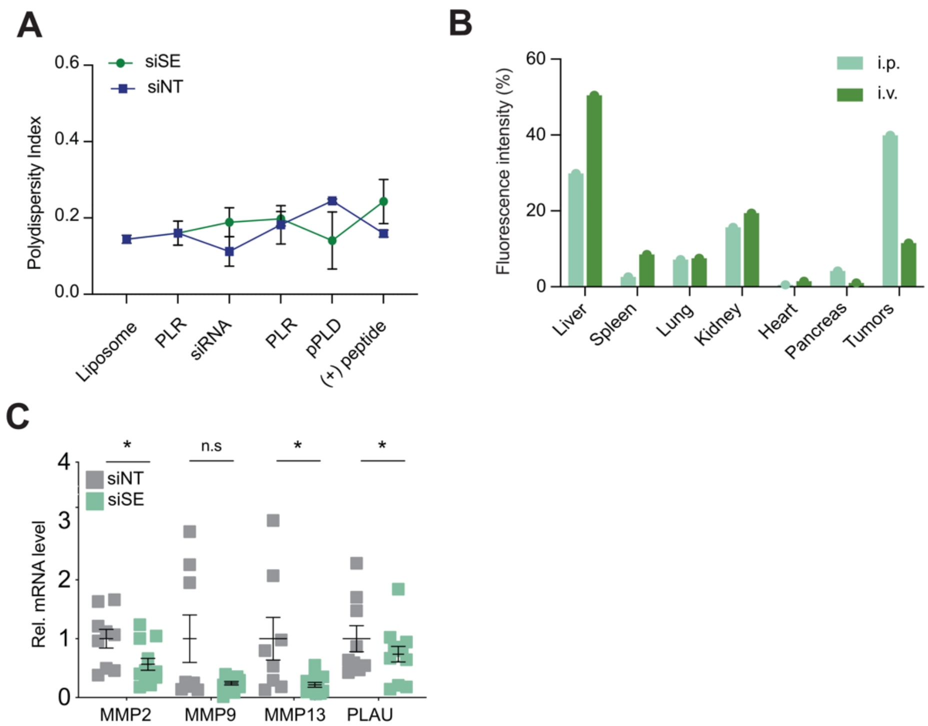

Fig S7. Characterization of theranostic LbL NPs *in vitro* and *in vivo*. (A) Polydispersity measurements of the LbL NPs were monitored during synthesis using dynamic light scattering. Error bars represent standard deviation of triplicate measurements. (**B**) Quantification of tissue fluorescence in Figure 5D. (**C**) Excised tumors were analyzed by qPCR for expression of proteases downstream of SMARCE1, with and without treatment with siSE (n=8 for siNT groups, n=9 for siSE groups; mean ± SEM, one-way ANOVA, *P<0.05).

Table S1. siRNA loading in LbL NPs.

siRNA in NP	Encapsulation Efficiency (%)	Weight % Loading	Peptide Conjugation (FAM)
siSMARCE1 (siSE)	57	34	1 ± 0.075
siSCRAMBLE (siNT)	72	43	0.95 ± 0.088

Table S2. List of proteases that cleave the substrates tested in 4E.

**Sequence**	**Top Proteases**					
PQGIWGQ	MMP13	FAP					
LVPRGSG	PRSS2	F2	CTSK	PCSK1			
PVGLIG	MMP13	MMP9					
PWGIWGQG	MMP13	MMP15	FAP	CTSK	CTSS		
PVPLSLVM	MMP3	MMP7	MMP10	MMP13			
PLGLRSW	MMP13	FAP	GZMA	KLK14	PRSS3		
fPRSGGG	KLK14	PRSS3	F2	HAT			
f-Pip-RSGGG	N/A						
**PLGVRGK**	MMP13	PRSS3	KLK14	MMP2	MMP9		
f-Pip-KSGGG	N/A						
GGSGRSANAK	PRSS3	KLK14	PLAU	HAT	HGFAC	LGMN	KLK5
ILSRIVGG	KLK14	PRSS2	PRSS1	MMP7	CTSS		
SGSKIIGG	KLK14	KLK11	PRSS3	PRSS2	PRSS1	CTSK	
GLGPKGQTG	PRSS3	MMP13	PRSS2	PRSS1	FURIN	ST14	

Table S3. List of all primary antibodies.

**Antibody**	**Catalog#**	**Manufacturer**	**Application**	**Dilution**
SMARCE1	ab70540	Abcam	IHC	1:200
MMP13	ab84594	Abcam	IHC	1:100
PLAU	ab24121	Abcam	IHC	1:100
Integrin alpha V	ab179475	Abcam	IHC	1:250

Table S4. Table of siRNA sequences used (sense).

**Scramble (siNT)**	UUC UCC GAA CGU GUC ACG U
**SMARCE1 (siSE)**	CCC AUA CCA GAA GAU GAG AAA
